# Family influences on physical activity and sedentary behaviours in Chinese junior high school students: a cross-sectional study

**DOI:** 10.1186/s12889-015-1593-9

**Published:** 2015-03-25

**Authors:** Xin Wang, Qing-Min Liu, Yan-Jun Ren, Jun Lv, Li-Ming Li

**Affiliations:** Department of Epidemiology and Biostatistics, School of Public Health, Peking University Health Science Centre, Beijing, 100191 China; Division for Chronic and Non-Communicable Disease Control and Prevention, Hangzhou Centre for Disease Control and Prevention, Mingshi Road, Jianqiao Town, Hangzhou, 310021 China; Division for Chronic and Non-Communicable Disease Control and Prevention, Hangzhou Centre for Disease Control and Prevention, Mingshi Road, Jianqiao Town, Hangzhou, 310021 China; Department of Epidemiology and Biostatistics, School of Public Health, Peking University Health Science Centre, Beijing, 100191 China; Department of Epidemiology and Biostatistics, School of Public Health, Peking University Health Science Centre, Beijing, 100191 China

**Keywords:** Moderate to vigorous intensity, Physical activity, Sedentary, Family influence, Adolescents

## Abstract

**Background:**

Family influence plays an important role in a child’s physical activity (PA). This study aimed to describe the level of moderate to vigorous intensity physical activity (MVPA) and sedentary behaviours among Chinese junior high school students and examine the associations between different types of family influence and MVPA or sedentary behaviours.

**Methods:**

Participants of two independent cross-sectional surveys, conducted in 2009 and 2011, were students in Grade 7 and 9 from all junior high schools in Hangzhou, China. The daily duration and frequency of MVPA, amount of sedentary time and frequency of family support were self-reported. Multi-level mixed-effects logistic regression was used to examine the associations between different types or levels of family influence and MVPA or sedentary behaviours.

**Results:**

A total of 7286 students were analysed finally. Overall, only 9.0% of the students participated in MVPA at least 60 minutes/day; 63.9% spent no more than 2 hours/day in sedentary behaviours. Frequent verbal encouragement and watching were associated with less leisure-time sedentary behaviours. The multivariate-adjusted odds ratios (ORs) for verbal encouragement and watching were 1.29 (95% CI, 1.08 to 1.55) and 1.19 (95% CI, 0.97 to 1.45) for 5-7 days per week. The involvement of family in the children’s activity in most days of the week was associated with both higher level of MVPA and less leisure-time sedentary behaviours. The respective ORs among students who reported familial support 5-7 days per week, were 1.50 (95% CI, 1.21 to 1.86) for engaging in seven days of MVPA per week, 1.67 (95% CI, 1.19 to 2.32) for at least 60 minutes of MVPA daily, and 1.48 (95% CI, 1.19 to 1.84) for no more than 2 hours of leisure-time sedentary behaviours daily.

**Conclusions:**

This study found that less than 10.0% of urban Chinese adolescents engaged in MVPA at least 60 minutes/day. Family involving themselves in the children’s activity exerted the most significant influence on children’s behaviours as compared with verbally encouraging and observing children’s activity. Any type of familial support including verbally encouraging, watching, and involving had effects on reducing leisure-time sedentary behaviours.

**Electronic supplementary material:**

The online version of this article (doi:10.1186/s12889-015-1593-9) contains supplementary material, which is available to authorized users.

## Background

Lack of physical activity (PA) and sedentary behaviours have been identified as risk factors for obesity, type 2 diabetes, cardiometabolic diseases, and depression [1-4]. Regular PA and reducing sitting time are associated with psychological benefits and better social development among adolescents. Also, physically active adolescents are more likely to avoid tobacco, alcohol, and drugs and therefore achieve better academic performance at school [5].An active lifestyle is not only necessary for physical and mental health in adolescence but also undoubtedly carried over into adulthood. Despite common knowledge on active lifestyle, a rapid decline in PA and an increase in sitting time have been observed in Chinese adolescents [6]. Over 50% of primary and secondary students in China spent less than one hour per day engaged in PA [7] and over 40% students spent more than two hours a day in screen-based activity [6].

Several studies have correlated PA of adolescents with their family support such as parents encouraging their children to be more physical active, watching their children engaging in PA, and involving themselves in their children’s activity [8-13]. These supports have been suggested to affect children’s behaviours differently [14]. However, instead of identifying their effects separately, previous studies mostly treated them as a whole.

Therefore, we aimed to (1) describe the levels of moderate to vigorous intensity physical activity (MVPA) and sedentary behaviours in a sample of junior high school students in Hangzhou of China; (2) examine the associations between different types of family support and MVPA and sedentary behaviours among Chinese adolescents. The data of this article were part of the data collected in a multinational collaboration programme of the Community Interventions for Health (CIH) [15]. The primary hypotheses in this analysis are that (1) family support is one of the most important motivational factors for children to move more and sit less; (2) children are motivated differently by different levels of support from verbally encouraging, watching, to involving.

## Methods

### Subjects and sampling methods

Participants were students in Grade 7 and 9 from all junior high schools in Xiacheng, Gongshu, and Xihu districts of Hangzhou City. Two independent cross-sectional surveys were conducted in 2009 and 2011 in the same schools. Both of surveys applied same sample size requirement and sampling design. The sample size estimation was based on the primary aim of CIH programme, that was, intervention group exposed to interventions regarding tobacco use, food choices, and PA had a 6% greater change in the prevalence of each of the three risk factors [15]. The final sample size was arrived at using knowledge of prevalence of the three risk factors in cities of China. The largest sample size across all three risk factors was selected as the necessary sample size. In addition, 20% was added to allow for non-response. The sample size for each of survey was 4800, with an equal size of 2400 subjects in grade 7 or 9. Stratified cluster sampling was used in both of the surveys. The list of classrooms was stratified by district, school, and grade. A simple random sample of classrooms were selected and all students in the selected classroom were eligible for the survey. Sampling fraction, that is, the ratio of sample size to population size, was applied to each strata to determine the number of classrooms and at least two classrooms in each strata were chosen. A total of 9271 students were investigated and the response rate was 94.8%. The study was approved by the Institutional Review Board at Peking University Health Science Centre (IRB00001052-08003). Informed consent ensuring privacy and confidentiality was obtained from both parent (or guardian) and student.

### Measures and variables

Each student completed the CIH Youth Survey questionnaire addressing knowledge of, attitudes to, and behaviours in relation to unhealthy diet, physical inactivity, and tobacco use [15]. CIH Youth Survey was designed based on the Health Behaviour in School-aged Children (HBSC) [16]. While formal reliability and validity survey were not conducted in this population, this questionnaire has been shown to have good reliability and validity in other Chinese children [17]. Structured questionnaires were self-administered to the students. Physical examinations including height and weight were measured by trained field staff. The definitions of variables analysed in this article and their grouping methods are described as follows.

### Assessment of PA and sedentary behaviours

The MVPA (including any activity that makes you sweat, get out of breath, and increases your heart rate, such as sports, school activities, playing with friends, and walking/cycling to or from school) was assessed using the following two questions: (1) How many days per week on average do you participate in MVPA (at least 30 minutes)(0 − 7 days); and (2) How many minutes per day on average do you participate in MVPA in the aforementioned days(30 − 39 minutes, 40 − 49 minutes, 50 − 59 minutes, or ≥ 60 minutes). According to global recommendations on PA, children and youth aged 5-17 should accumulate at least 60 minutes of MVPA daily [18]. Therefore, the frequency (<7 days/week or 7 days/week) and duration (<60 minutes/day or ≥ 60 minutes/day) of MVPA were dichotomised. In addition, the total level of MVPA per week was dichotomized as follows: (1) meeting the recommendation: engaging in at least 60 minutes of MVPA daily; or (2) not meeting the recommendation: less than 7 days of MVPA per week or less than 60 minutes of MVPA per day. Sedentary behaviours were assessed using two questions as follows: (1) on school days, how much time per day on average do you spend in sedentary behaviours for leisure, including using a computer for pleasure, watching television/videos, playing electronic games, sitting and chatting, or other sitting activities (0 hours/day − ≥7 hours/day); and (2) on weekends, how much time per day on average do you spend in sedentary behaviours for leisure(0 hours/day − ≥7 hours/day). According to the global guidelines on youth sedentary behaviour, youth aged 12-17 should limit recreational screen time to no more than 2 hours per day [19]. Thus, the amount of sedentary time on school days and weekends was dichotomized as follows: (1) meeting the recommendation: no more than 2 hours of leisure-time sedentary behaviours per day; or (2) not meeting the recommendation: more than 2 hours of leisure-time sedentary behaviours per day.

#### Assessment of family support

Family support was assessed by the following three questions: (1) how many days per week on average do your families encourage you to engage in PA; (2) how many days per week on average do your families watch you engaging in PA; and (3) how many days per week on average do your families involve themselves in your activities, that is, having PA together with you. Response options included: (1) never or less than weekly; (2) 1-2 days/week; (3) 3-4 days/week; (4) 5-6 days/week; and (5) 7 days/week. The last two options were combined in the analyses.

#### Assessment of other covariates

Sex, grade (grade 7 or grade 9), mother’s level of education (junior high school or below, senior high school, college or above, or don’t know), health belief, and their perception of the neighbourhood environment, were reported by all students. BMI was calculated as weight in kilograms divided by the square of height in meters. Health belief was assessed by the following question: “To stay healthy, at least how much time per day should children like your age engage in PA” (15 minutes, 30 minutes, 60 minutes, 90 minutes, or do not know/not sure). We grouped health belief into two categories: (1) less than 60 minutes or do not know/not sure; or (2) at least 60 minutes. The perceptions of the neighbourhood environment were assessed by the following three questions: (1) it is unsafe to walk or jog because of the busy traffic and no walking trails around my neighbourhood; (2) parks or gyms are easily accessible around my neighbourhood; and (3) it was safe to walk or jog around my neighbourhood during the day. Response options for these three questions were as follows: (1) disagree strongly, (2) disagree slightly, (3) not sure, (4) agree slightly, or (5) agree strongly. We grouped the perceptions of the neighbourhood environments into three categories (agree, disagree, or not sure) in the analyses.

### Statistical analyses

Chi-square tests were used to compare the counts of categorical responses between two or more independent groups. Nonparametric tests of K-independent samples were performed on the equality of the medians for the continuous variables without normal distribution. Statistical significance was defined as P < 0.05. The statistical analyses were performed with Stata 12.0 (StataCorp LP, 4905 Lakeway Drive, College Station, TX 77845 USA).

Three-level nested models were specified (individual, class, and school) using the xtmelogit commands to address the dependency between the individuals in the cluster sampling data. Four dependent variables were tested:(1) participating in seven days of MVPA per week (yes/no); (2) at least 60 minutes of MVPA on days in which students reported having MVPA (yes/no); (3) meeting the recommended level of at least 60 minutes of MVPA daily; and (4) meeting the recommendation of no more than 2 hours of leisure-time sedentary behaviours per day (yes/no). Multivariate models were fitted with different levels of adjustment. Model 1 included each type of family support (i.e., verbally encouraging, watching, or involving) separately. Model 2 included three types of family support simultaneously. Model 3 additionally included sex, grade, BMI, and health belief. Model 4 additionally included survey time (2009 or 2011), mother’s level of education, and the perceptions of the neighbourhood environment. The tests for linear trend across familial support categories were performed by assigning the midpoint values of each frequency category and treating the variable as continuous in a separate model. We also examined associations between family support and MVPA and sedentary time across gender. Test for interaction were performed by means of likelihood ratio tests to compare multivariate models with and without the interaction terms between family support and gender.

## Results

We excluded students who were unable to engage in PA due to disability, temporary or permanent disease (n = 895) and with missing in any variables (n = 1090). The final analyses included 3811 (52.3%) boys and 3475 (47.7%) girls. As compared with the excluded students, students kept in the analyses were more in Grade 7 and with more highly-educated mother. There was no statistically significant difference in sex and BMI. Overall, 31.2% of students participated in seven days of MVPA per week and 14.6% had at least 60 minutes of MVPA on days in which students reported having MVPA. Only 9.0% of students met the recommended level of PA for children − that is, at least 60 minutes of MVPA daily. 63.9% of students met the recommendation of no more than 2 hours of leisure-time sedentary behaviours per day, with a higher proportion on school days than on weekends. Higher proportions of active students, those who participated in seven days of MVPA per week, had at least 60 minutes of MVPA on days in which they reported having MVPA, met the recommended level of PA and met the recommendation of no more than 2 hours of leisure-time sedentary behaviours per day, received family support almost every day (P value for trend < 0.05, Table 1). The characteristics of the study participants according to familial support were presented in the Table 1.

**Table 1:** Characteristics by family influence group (N = 7286)*

	**Verbal encouragement (days/week)**					**Watching (days/week)**					**Involvement (days/week)**				
	**Less than weekly**	**1-2**	**3-4**	**5-7**	**P**	**Less than weekly**	**1-2**	**3-4**	**5-7**	**P**	**Less than weekly**	**1-2**	**3-4**	**5-7**	**P**
N	878	1670	1417	3321		2617	2527	1028	1114		2643	2829	926	888	
**Grade, n (%)**															
Grade 7															
	359 (40.9)	770(46.1)	684 (48.3)	1901 (57.2)	<0.001	1083 (41.4)	1345 (53.2)	612 (59.5)	674 (60.5)	<0.001	1037 (39.2)	1523 (53.8)	576 (62.2)	578 (65.1)	<0.001
Grade 9															
	519 (59.1)	900(53.9)	733 (51.7)	1420 (42.8)		1534 (58.6)	1182 (46.8)	416 (40.5)	440 (39.5)		1606 (60.8)	1306 (46.2)	350 (37.8)	310 (34.9)	
**Mother’s level of education, n (%)**															
Low															
	319 (36.3)	552 (33.1)	421 (29.7)	848 (25.5)	<0.001	903 (34.5)	709 (28.1)	248 (24.1)	280 (25.1)	<0.001	912 (34.5)	752 (26.6)	247 (26.7)	229 (25.8)	<0.001
Middle															
	218 (24.8)	403 (24.1)	358 (25.3)	763 (23.0)		644 (24.6)	616 (24.4)	232 (22.6)	250 (22.4)		667 (25.2)	663 (23.4)	210 (22.7)	202 (22.7)	
High															
	295 (33.6)	641 (38.4)	589 (41.6)	1575 (47.4)		948 (36.2)	1103 (43.6)	506 (49.2)	543 (48.7)		946 (35.8)	1302 (46.0)	431 (46.5)	421 (47.4)	
Not sure															
	46 (5.2)	74 (4.4)	49 (3.5)	135 (4.1)		122 (4.7)	99 (3.9)	42 (4.1)	41 (3.7)		118 (4.5)	112 (4.0)	38 (4.1)	36 (4.1)	
**BMI (kg/m** ^**2**^ **),** $\overline{\mathrm{X}}\pm \mathrm{S}\mathrm{d}$															
	20.0 ± 3.0	19.9 ± 3.2	20.1 ± 3.3	20.3 ± 3.6	0.127	20.3 ± 3.4	20.0 ± 3.3	20.1 ± 3.5	20.0 ± 3.5	0.003	20.3 ± 3.3	20.0 ± 3.4	20.0 ± 3.5	19.9 ± 3.4	<0.001
**MVPA status, n (%)**															
7 days/wk															
	283(32.2)	364(21.8)	365(25.8)	1260(37.9)	<0.001	776(29.7)	642(25.4)	313(30.4)	541(48.6)	<0.001	770(29.1)	782(27.6)	280(30.2)	440(49.5)	<0.001
At least 60 minutes/day															
	168(19.1)	172(10.3)	169(11.9)	557(16.8)	<0.001	419(16.0)	278(11.0)	131(12.7)	238(21.4)	0.007	422(16.0)	341(12.1)	104(11.2)	199(22.4)	0.001
At least 60 minutes/day, 7 days/wk															
	99(11.3)	86(5.1)	92(6.5)	376(11.3)	<0.001	246(9.4)	147(5.8)	75(7.3)	185(16.6)	<0.001	247(9.3)	182(6.4)	60(6.5)	164(18.5)	<0.001
**Sedentary behaviours (no more than 2 hours), n (%)**															
On school days															
	721(82.1)	1445(86.5)	1250(88.2)	3019(90.9)	<0.001	2266(86.6)	2252(89.1)	915(89.0)	1002(89.9)	0.004	2286(86.5)	2537(89.7)	813(87.8)	799(90.0)	0.015
On weekends															
	296(33.7)	614(36.8)	497(35.1)	1409(42.4)	<0.001	905(34.6)	989(39.1)	395(38.4)	527(47.3)	<0.001	906(34.3)	1091(38.6)	385(41.6)	434(48.9)	<0.001
During the week															
	638(72.7)	1354(81.1)	1170(82.6)	2881(86.8)	<0.001	2073(79.2)	2125(84.1)	880(85.6)	965(86.6)	<0.001	2081(78.7)	2408(85.1)	784(84.7)	770(86.7)	<0.001
**Health belief (at least 60 minutes/day), n (%)**															
	293(33.4)	533(31.9)	435(30.7)	1313(39.5)	<0.001	903(34.5)	808(32.0)	397(38.6)	466(41.8)	<0.001	944(35.7)	957(33.8)	322(34.8)	351(39.5)	0.071
**Opinion of neighbourhood environment, n (%)**															
Busy traffic															
	114(13)	188(11.3)	140(9.9)	286(8.6)	<0.001	304(11.6)	256(10.1)	81(7.9)	87(7.8)	<0.001	296(11.2)	287(10.1)	74(8.0)	71(8.0)	<0.001
Easily available parks or gyms															
	499(56.8)	998(59.8)	927(65.4)	2385(71.8)	<0.001	1564(59.8)	1678(66.4)	726(70.6)	841(75.5)	<0.001	1602(60.6)	1870(66.1)	654(70.6)	683(76.9)	<0.001
Safe during the day															
	594(67.7)	1172(70.2)	1043(73.6)	2614(78.7)	<0.001	1860(71.1)	1896(75)	794(77.2)	873(78.4)	<0.001	1875(70.9)	2133(75.4)	72478.2)	691(77.8)	<0.001

*Watching: Families watched the child participate in PA. Verbal encouragement: Families encouraged the child to do PA. Involvement: Families involved the child in PA. MVPA: Moderate to vigorous intensity physical activity; BMI: Body Mass Index; Sd: Standard deviation; X̅: Mean. Family influence was recoded as the continuous variable: (1) less than weekly: 0; (2) 1-2 days/week: 1.5; (3) 3-4 days/week: 3.5; (4) 5-7 days/week: 6. P: P-values for trend represent family influence group comparison.

**Figure 1:**
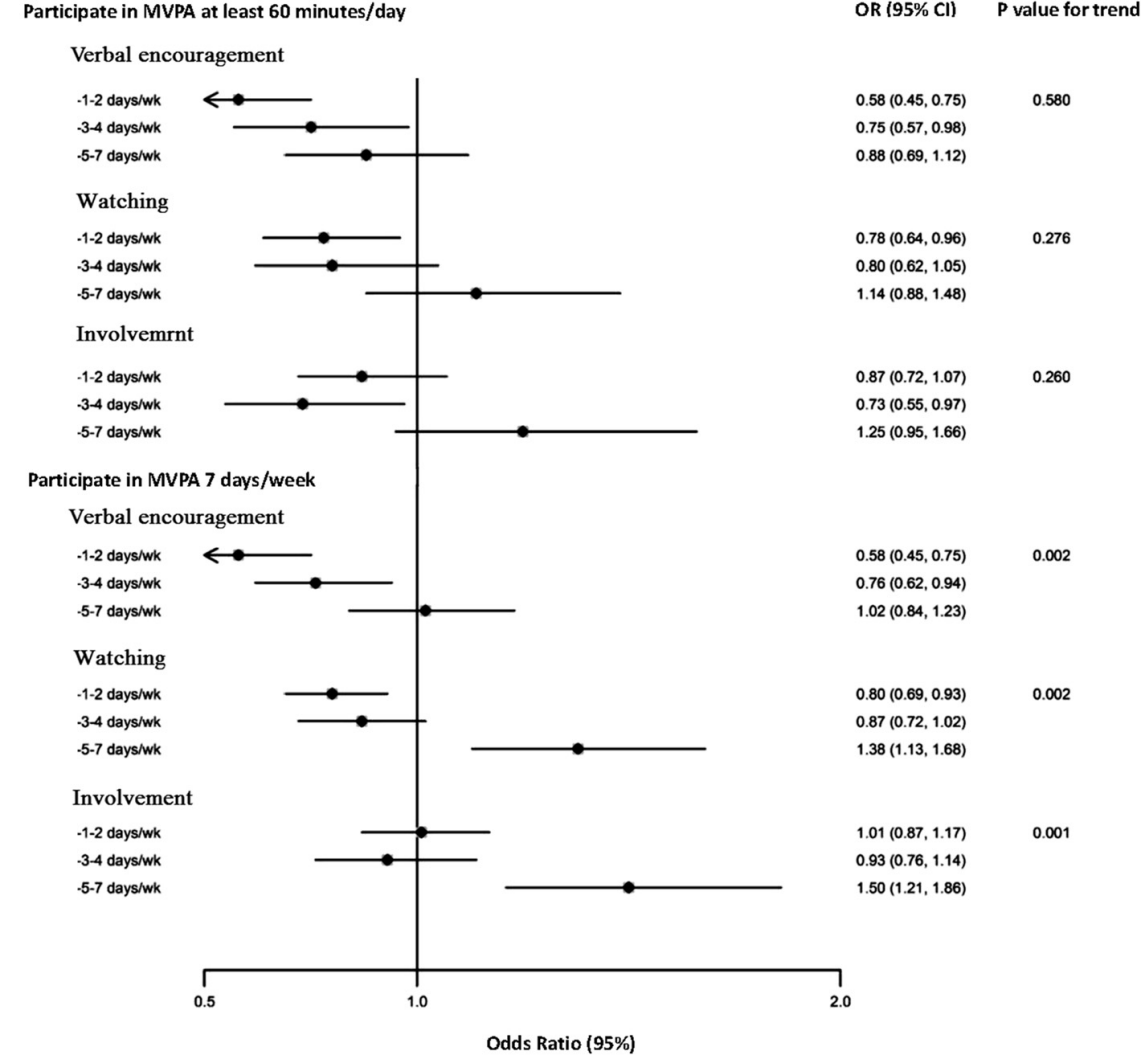
**Odds ratios** **(ORs)** **for the daily duration and the frequency of child’** **s MVPA compared with recommendations by family influence.** Adjusted for sex, grade, BMI, health belief, survey time, mother’s level of education and their opinions regarding the neighbourhood environment. Dots represent the ORs. Horizontal lines represent the corresponding 95% CIs. Watching: Families watched the child participate in PA. Verbal encouragement: Families encouraged the child to do PA. Involvement: Families involved the child in PA. P: P-values for trend.

**Figure 2:**
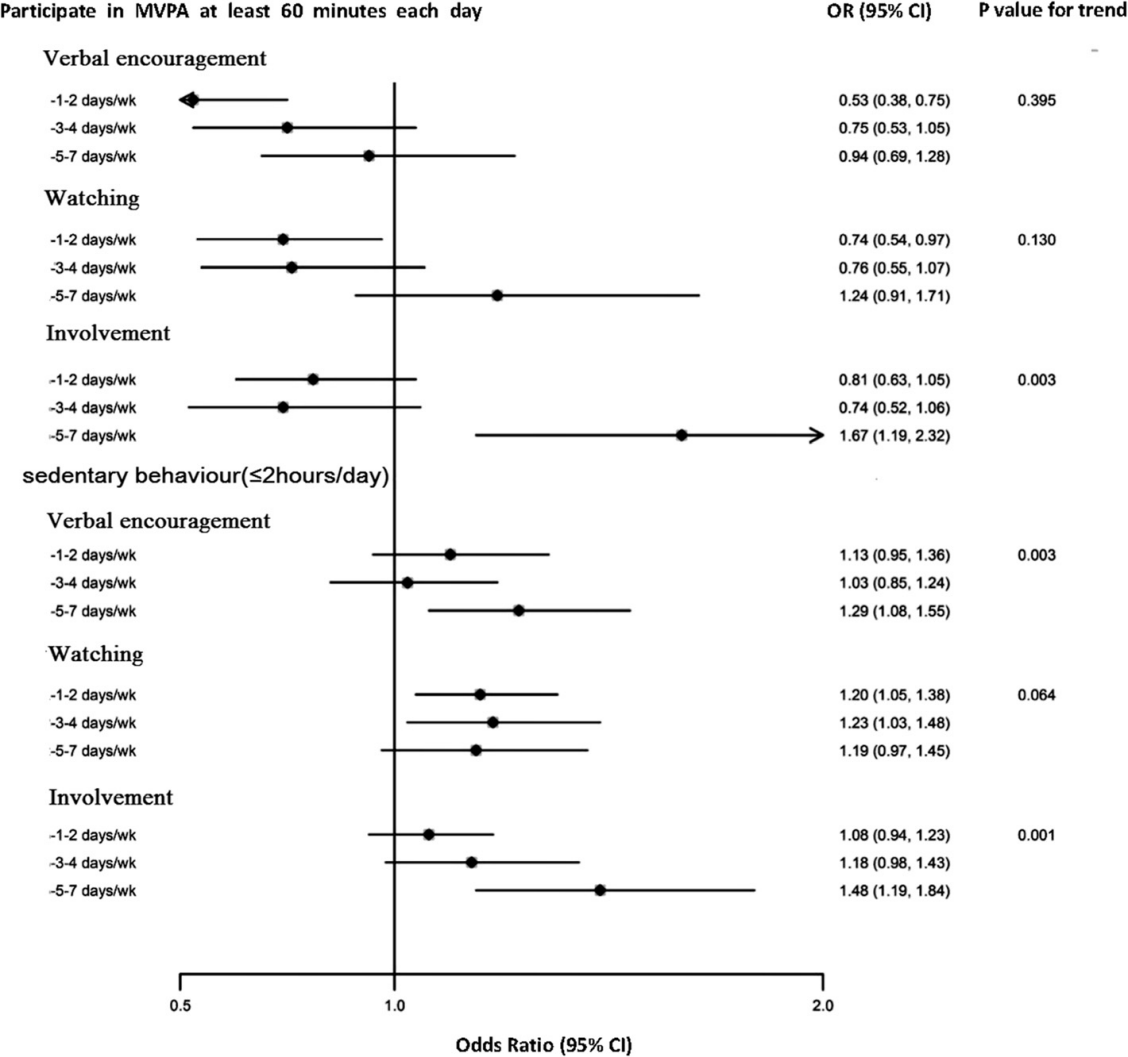
**Odds ratios (ORs) for total MVPA level and sedentary behaviours of child compared with recommendations by family influence.** Adjusted for sex, grade, BMI, health belief, survey time, mother’s level of education and their opinions regarding the neighbourhood environment. Dots represent the ORs. Horizontal lines represent the corresponding 95% CIs. Watching: Families watched the child participate in PA. Verbal encouragement: Families encouraged the child to do PA. Involvement: Families involved the child in PA. P: P-values for trend.

Multivariate-adjusted analyses showed that neither frequent verbal encouragement nor frequent watching were associated with higher level of MVPA (Figures 1 and 2). Students who reported familial support 1-2 days per week had lower frequency and duration of PA instead as compared with those who reported familial support less than once per week. However, frequent verbal encouragement and watching were associated less leisure-time sedentary behaviours. As compared with students who reported familial support less than once per week, the multivariate-adjusted odds ratios (ORs) for verbal encouragement were 1.13 (95% CI, 0.95 to 1.36) for 1-2 days per week, 1.03 (95% CI, 0.85 to 1.24) for 3-4 days per week, and 1.29 (95% CI, 1.08 to 1.55) for 5-7 days per week; the respective ORs for watching were 1.20 (95% CI, 1.05 to 1.38), 1.23 (95% CI, 1.03 to 1.48), and 1.19 (95% CI, 0.97 to 1.45). More details were shown in Additional file 1: Table S1-S4.

Multivariate-adjusted analyses showed that the involvement of family member in the children’s activity in most of the days of the week was associated with both greater frequency and longer duration of MVPA and less leisure-time sedentary behaviours. The multivariate-adjusted ORs among students who reported familial support 5-7 days per week, as compared with students who reported less than once per week, were 1.25 (95% CI, 0.95 to 1.66) for at least 60 minutes of MVPA on days in which students reported having MVPA, 1.50 (95% CI, 1.21 to 1.86) for engaging in seven days of MVPA per week, 1.67 (95%CI, 1.19 to 2.32) for meeting the recommended level of at least 60 minutes of MVPA daily, and 1.48 (95%CI, 1.19 to 1.84)for no more than 2 hours of leisure-time sedentary behaviours per day (Figures 1 and 2). More details were shown in Additional file 1: Table S1-S4.

## Discussions

In this large sample size study of Chinese urban adolescents, we found that less than 10.0% of students met the recommended level of PA for children − that is, at least 60 minutes of MVPA daily. This study is also one of the few studies to explore the relationship between the family support and children’s PA and sedentary behaviours in Chinese urban adolescents. We found that family members involving themselves in the children’s activity had the most significant influence on children’s behaviours as compared with other types of supports such as verbally encouraging and observing children’s activity. Involvement of family members in the children’s activity almost every day was associated with higher level of MVPA and less leisure-time sedentary behaviours.

It has been suggested that strong family support increases children’s self-efficacy to overcome barriers to being physically active [8]. Self-efficacy refers to a person’s belief in his or her ability to succeed in a particular situation [20]. Children who have strong self-efficacy believe that they can successfully overcome obstacles to physical fitness and skills. Persuasive information, as in verbal encouragement from parents, can raise self-efficacy, but its effects can be transitory if subsequent performance turns out differently [21]. In contrast with verbally expressed expectation, the effects of parental expectations on children’s behaviours seem greatest when a high level of parent involvement exists [21]. The familial involvement in PA may boost children’s efficacy to improve MVPA, help children develop skills necessary to be physically active, and provide logistical support [8,22].

Sedentary behaviours have emerged as an important health risk factor independent of MVPA [23,24]. Although verbally encouraging and watching were not associated with a higher level of MVPA in this study, they were observed to be associated with less leisure-time sedentary behaviours. One possible explanation is that some bouts of light activity have replaced sedentary time under the familiar pressure on children. Several previous studies have reported that sedentary time was inversely correlated with light-intensity PA such as walking and playing [25,26]. While increasing MVPA should still be a public health priority, breaks in sedentary time and light-intensity PA may also provide a beneficial adjunct to the current 60 min/day of MVPA recommendation [27,28].

In the current study, we hypothesised that familial support such as verbal encouragement, watching, and involvement is one of the most important motivations for children to get outdoors in the leisure time at home. The more frequent the family members are concerned about their children’s physical activity in whatever way, the more physically active children are. We used the weekly frequency as a measurement for the strength of familial support. Familial support could also manifest itself in other ways. For example, parents watch the exercise sessions which they book for their children or involve themselves in planned school events. In this situation, the proportion of parents attending scheduled events would be a better measurement for the strength of familial support.

This study acknowledges a few limitations that should be noted. First, given the cross-sectional nature of this study, it is not possible for us to determine the temporal relationship between familial support and children’s behaviours. Two-way causal relationship might exist. Stronger family support is associated with more physically active children, while for physically active children who like and specialize in sports, parents may save the efforts to push their children into playing sports. In the current study, we could not exclude the possibility that some physically active children consequently received less familial support which might have attenuated the positive influence of familiar support on children’s behaviours toward null. Second, participants were more likely from educated households, possibly resulting in a potential bias [13]. However, the lack of family involvement in children’s PA who were from educated household highlights the importance of increasing family involvement to promote children’s MVPA and to reduce sedentary time. Third, residual confounding by other unmeasured or unknown factors such as children’s preference for PA and parental level of PA remains possible although we have carefully adjusted for some potential factors for children’s PA. Forth, the levels of MVPA, sedentary time, and family support were self-reported; therefore, some measurement error is inevitable. However, relevant survey instruments have been shown to have good reliability and validity in diverse populations [17,29-31].

## Conclusion

In summary, in this large sample size study of Chinese urban adolescents, we found that family members involving themselves in the children’s activity had the most significant influence on children’s behaviours as compared with other types of supports such as verbally encouraging and observing children’s activity. Any type of familial support including verbally encouraging, watching, and involving had effects on reducing leisure-time sedentary behaviours.

## Additional file

Additional file 1: Table S1. The association between family influence and the duration of MVPA per day (n = 7286). OR (95% CI) for the duration of MVPA of child (at least 60 minutes/day or not) by family influence. Multivariate models were adjusted for the following factors: (1) model 1 included certain family influence (encourage, watch or involve in); (2) model 2 included all family influence (encourage, watch and involve in); (3) model 3 additionally included sex, grade, BMI and health belief of daily PA time; (4) model 4 additionally included survey time, mother’s level of education and opinion regarding neighbourhood environment. Based on model 4, three-level nested models were specified (individual, class and school) with equal random effects variance and covariance being zero, and the coefficient is the SD (95% CI) of random intercept. Family influence was recoded as the continuous variable: (1) less than weekly: 0; (2) 1-2 days/week: 1.5; (3) 3-4 days/week: 3.5; (4) 5-7 days/week: 6. P-values for trend represent family influence group comparison. **Table S2.** The association between family influence and the frequency of MVPA (n = 7286). OR (95% CI) for the frequency of MVPA of child (7 days/week or not) by family influence. **Table S3.** The association between family influence and total MVPA per week (n = 7286). OR (95% CI) for the total level of MVPA of child (at least 60 minutes each day or not) by family influence. **Table S4.** The association between family influence and sedentary time (n = 7286). OR (95% CI) for sedentary time of child (at least 60 minutes/day or not) by family influence.

## Abbreviations

MVPA: Moderate to vigorous intensity physical activity
PA: Physical activity
BMI: Body mass index
Sd: Standard deviation
X̅: Mean
OR: Odds ratio
CI: Confidence interval
Xtmelogit: Multilevel mixed-effects logistic regression
